# Mutational drivers of cancer cell migration and invasion

**DOI:** 10.1038/s41416-020-01149-0

**Published:** 2020-11-18

**Authors:** Nikita M. Novikov, Sofia Y. Zolotaryova, Alexis M. Gautreau, Evgeny V. Denisov

**Affiliations:** 1 Laboratory of Cancer Progression Biology, Cancer Research Institute, Tomsk National Research Medical Center, Russian Academy of Sciences, Tomsk, Russia; 2 CNRS UMR7654, Ecole Polytechnique, Institut Polytechnique de Paris, Palaiseau, France; 3 School of Biological and Medical Physics, Moscow Institute of Physics and Technology, Dolgoprudny, Russia

**Keywords:** Cancer genetics, Cancer genomics

## Abstract

Genomic instability and mutations underlie the hallmarks of cancer—genetic alterations determine cancer cell fate by affecting cell proliferation, apoptosis and immune response, and increasing data show that mutations are involved in metastasis, a crucial event in cancer progression and a life-threatening problem in cancer patients. Invasion is the first step in the metastatic cascade, when tumour cells acquire the ability to move, penetrate into the surrounding tissue and enter lymphatic and blood vessels in order to disseminate. A role for genetic alterations in invasion is not universally accepted, with sceptics arguing that cellular motility is related only to external factors such as hypoxia, chemoattractants and the rigidity of the extracellular matrix. However, increasing evidence shows that mutations might trigger and accelerate the migration and invasion of different types of cancer cells. In this review, we summarise data from published literature on the effect of chromosomal instability and genetic mutations on cancer cell migration and invasion.

## Background

**Fig. 1:**
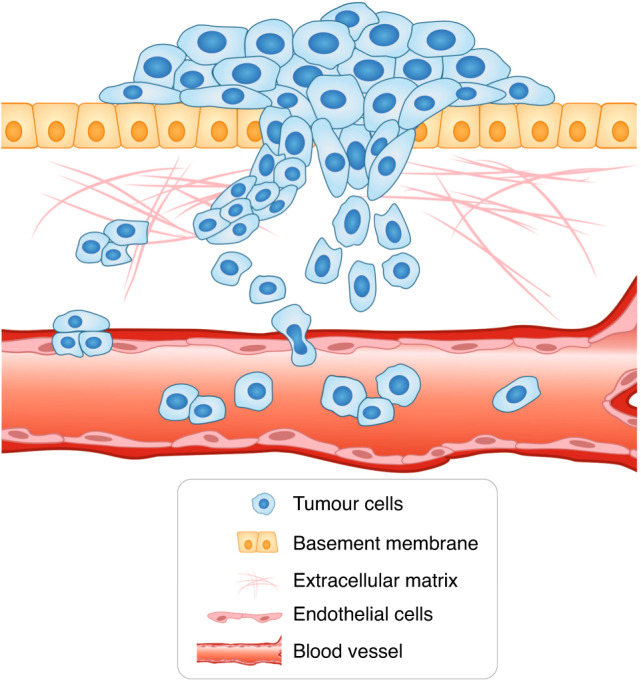
The model of cancer cell invasion. Cancer invasion is the first step of the metastatic cascade. Tumour cells penetrate the basement membrane and invade the surrounding tissues using two modes of movement—individual and collective invasion. Invading tumour cells reach the blood vessel, enter the blood flow and disseminate, eventually giving rise to secondary tumours.

Genetic abnormalities lie at the heart of most cancers—mutations can transform normal cells into cancerous ones by endowing them with new properties. Genome instability and mutations determine the hallmarks of cancer, one of which is the ability of tumour cells to invade and metastasise.^1^ Metastasis is the leading cause of death from cancer. During the process of metastasis, tumour cells leave the primary site and spread throughout the body, forming secondary sites and causing severe organ failure.^2^ The first step of the metastatic cascade is invasion, in which tumour cells penetrate their surrounding basement membrane and migrate through the extracellular matrix (ECM) into the surrounding tissue (Fig. 1).^3^

Several different parameters in the tumour microenvironment influence the regulation of cancer cell migration and invasion: the presence of hypoxia, chemoattractants, ECM stiffness and a lack of nutrients prompt cancer cells to start searching for a ‘better life’.^4^ Of particular significance during migration and invasion is the phenomenon of epithelial-to-mesenchymal transition (EMT), which determines the plasticity of tumour cells, allowing them to switch from a non-motile epithelial to a motile mesenchymal state, and endowing cancer cells with multiple malignant features, such as the increased invasiveness and resistance to senescence, apoptosis and treatment.^2^ The EMT is activated by transcription factors, such as Twist, Snail, Slug and Zeb1, through various signalling pathways, the most important being TGF-β, WNT and Notch pathways.^5^ The availability of these transcription factors can therefore offer a means of regulating this reversible and plastic process, with control also occurring at epigenetic and post-translational levels.^5^ The impact of somatic mutations incurred during primary tumour formation on EMT remains to be elucidated.^2^

The role of genetic alterations in tumour cell migration and invasion has received undeservedly little attention compared with epigenetic and transcriptional mechanisms of cell motility. Despite the huge amount of experimental data regarding the effect of genetic mutations on cancer invasion, only a few reviews exist, most of which focus mainly on the tumour suppressor p53.^6,7^ In this review, we summarise published data outlining chromosomal instability (CIN) and gene alterations that impinge on some of the molecular components that are crucial for cancer cell migration and invasion. We also discuss the main difficulties encountered in identifying genetic alterations that drive cancer invasion and suggest potential models and approaches to overcome these problems. Finally, we underscore the significance of identifying mutational drivers of cancer invasion as potential therapeutic targets for the prevention of metastatic disease.

## Chromosomal instability

CIN, which includes changes in the number of chromosomes as well as their rearrangement, is observed in many tumour types and is associated with tumour progression, as described in Box 1.^8^ For example, as shown in MDA-MB-231 triple-negative breast cancer cells in vitro and in vivo, CIN can induce the transcriptional transition of tumour cells to a mesenchymal state characterised by increased migratory and invasive behaviour with the activation of inflammatory pathways.^9^ By increasing inflammation, CIN can also promote cancer metastasis.^9,10^ It is worth noting, however, that CIN can influence the invasive and metastatic potential differently, depending on the molecular landscape of tumour cells and their microenvironment (reviewed in ref. ^10^).

**Fig. 2:**
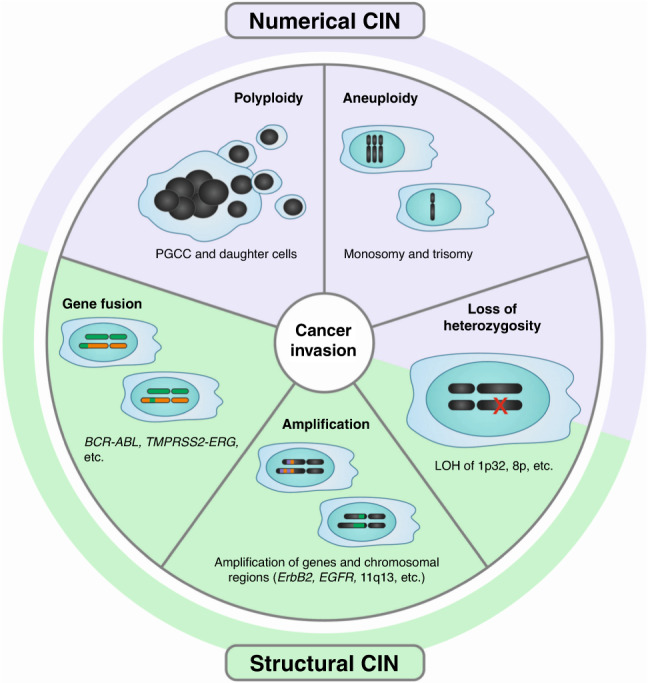
Chromosomal instability and cancer invasion. Chromosomal instability (CIN) is one of the cancer hallmarks and plays an important role in tumour cell migration and invasion. CIN can be represented by gain or loss of whole chromosomes (numerical CIN) and chromosomal rearrangements (structural CIN). Loss of heterozygosity (LOH) that can be attributed to numerical and structural CIN simultaneously, depending on the type of genomic changes resulting in the allele loss, affects the invasive potential of tumour cells. Polyploidy defined as the presence of additional sets of chromosomes drastically changes the genetic landscape of tumour cells, endowing them with high invasive potential. Polyploid giant cancer cells (PGCCs) are found in various cancers and show extreme tumorigenic, invasive and metastatic potential. Aneuploidy when chromosomes can be lost (monosomy) or gained (trisomy) can have different effects on tumour cell invasion: from attenuation of migratory behaviour to its enhancement. Different gene fusions arising from various chromosomal rearrangements affect tumour cell motility through diverse signalling pathways and mechanisms. Amplification defined as a copy number increase of a certain region of the genome leads to enhanced gene expression and, if a gene positively regulates cellular motility, it can accelerate cancer invasion.

Two types of CIN can be distinguished (Fig. 2): numerical CIN, which is determined by the gain or loss of whole chromosomes (aneuploidy) and chromosome sets (polyploidy), and structural CIN, which involves fractions of chromosomes and can result in gene fusions, amplifications and other alterations.^8^ In both cases, loss of heterozygosity (LOH)—defined as the loss of one allele caused by deletion, mitotic recombination, gene conversion or loss of a chromosome—can arise.^11^ LOH is a common alteration in cancer; it results in haploinsufficiency or loss of gene expression, and frequently affects tumour-suppressor genes, thereby contributing to tumorigenesis. In addition, LOH—alone or together with other genetic or epigenetic alterations—can influence the ability of cancer cells to invade.^12,13^ For example, LOH of the 8p22 chromosomal region (*DLC1*, which encodes a Rho GTPase-activating protein) promotes migration and invasion of breast,^14^ lung,^15^ prostate^16^ and liver^17^ cancer cells in vitro.^18^ LOH of the 8p region leads to changes in lipid metabolism, which, in turn, increases the motility and invasiveness of MCF10A breast cells in vitro.^19^ Loss of the expression of *TGFBR3*, which encodes TGF-βR3, due to LOH of the 1p32 region, enhances migration and invasion of A549 non-small-cell lung cancer (NSCLC) cells in vitro.^20^

### Numerical CIN

Gain or loss of whole chromosomes (aneuploidy) or chromosome sets (polyploidy) are frequent events in various cancers and can drastically affect tumour progression not only through transcriptomic changes but also through the enhancement of CIN itself, creating more and more genetically distinct cancer cell clones.^8^

It is believed that the polyploidisation of tumour cells is only a step on the path to aneuploidy.^21,22^ However, polyploid tumour cells can exist without transitioning to aneuploidy.^21^

Polyploid tumour cells contribute significantly to cancer progression. Polyploid giant cancer cells (PGCCs) are formed by endoreplication or fusion of several cells, and are found in high-grade and chemoresistant cancers, predominantly in breast, ovarian and colorectal cancers.^23,24^ PGCCs can survive anticancer therapy, are extremely tumorigenic and contribute to cancer metastasis.^23,24^ PGCCs and their daughter cells, collectively called tumour buds and located at the invasive front of tumours,^25^ have a mesenchymal phenotype and a high capacity for invasion through changes in the expression of factors that mediate EMT.^26–28^ In the MDA-MB-231 breast cancer cell line, PGCCs moved more slowly than normal cancer cells, but showed high migratory persistence.^29^ This migratory phenotype is associated with the dysregulation of the actin network and RhoA–Rho-associated protein kinase (ROCK)1 signalling pathway, which drives increased cell stiffness.^29^ As shown in LoVo and HCT116 colorectal cancer cells in vitro and in vivo, the migration and invasion of PGCCs and their daughter cells might be determined by S100A4 and its associated molecular network, potentially involving regulation of the structure and function of the annexin A2–S100A10 complex to influence cathepsin B, as well as cytoskeletal associations with 14–3–3 ζ/δ and ezrin.^30^ In addition to PGCCs, other polyploid cells can contribute to tumour metastasis. For example, as shown in the DLD-1 cell line, tetraploid tumour cells observed at the invasive front of colorectal adenocarcinomas are characterised by an enhanced capability to migrate and invade.^31^

Aneuploidy has long been known to be associated with an increased expression of genes related to EMT, cancer cell migration, invasion and metastasis.^32^ However, different aneuploidies have distinct effects on cancer cell invasion.^33^ For example, DLD-1 colorectal cancer cells with trisomy of chromosome 7 or 13 invade more actively than diploid cells, both in standard and stressful conditions (hypoxia, etc.) in vitro.^34^ Similarly, trisomy of chromosome 5 enhances the invasive potential of HCT116 colorectal cancer cells in vitro and in vivo through partial EMT and upregulation of matrix metalloproteinases (MMPs).^33^ By contrast, trisomy of chromosome 13 or 18 significantly decreases invasion of HCT116 colorectal cancer cells in vitro, potentially because of aneuploidy-induced dosage imbalances that may interfere with different cellular functions, including cell motility.^33^

### Structural CIN

Chromosomal rearrangements can lead to the loss of tumour suppressors and/or the amplification of oncogenes and can contribute to cancer progression.

Gene fusions are a frequent result of chromosomal rearrangements and can result from translocations, deletions, inversions and duplications, as well as chromothripsis, a catastrophic genomic event leading to massive rearrangements of multiple chromosomes.^35^ Owing to the large number of gene fusions, their role in cancer cell migration and invasion could be the topic of another review, so we consider here some of the most common gene fusions. The first gene fusion to be discovered, *BCR–ABL*, is the result of a reciprocal translocation between chromosomes 22 and 9, and is detected in >96% of patients with chronic myeloid leukaemia.^35^ This fusion causes alterations in the actin cytoskeleton that promote the motility of chronic myeloid leukaemia cells, as demonstrated in various cell lines in vitro.^36,37^ The *TMPRSS2–ERG* gene fusion can arise from the inversion or interstitial deletion of chromosome 21q22 and is found in 50% of prostate cancers.^35^ This gene fusion leads to the overexpression of *ERG* (ETS-related gene), a transcription factor, which, in turn, promotes prostate tumour cell movement through Notch signalling or transcriptional activation of MMP9 and plexin A2, a semaphorin co-receptor.^38–40^ ERG overexpression as a result of the *TMPRSS2–ERG* gene fusion event has been demonstrated to promote EMT not only by activating TGF-β signalling but also by inducing WNT signalling.^41,42^ Other gene fusions also contribute to EMT. The *MLL–AF9* translocation *t*(9;11) is found in acute myeloid leukaemia and promotes tumour invasion associated with the transcription factor ZEB1 in a long-term haematopoietic stem-cell-derived mouse model of acute myeloid leukaemia.^43^ Fusions between the oestrogen receptor gene (*ESR1*) and *YAP1* (which encodes Yes1-associated transcriptional regulator) or *PCDH11X* (which encodes the cell adhesion protein protocadherin 11 X-linked) are associated with the induction of EMT and were shown to enhance the motility of T47D breast cancer cells in vitro and the metastasis of T47D xenografts.^44^

Gene amplifications are frequently occurring events in many cancers and result in overexpression of genes—mainly oncogenes—that confer a growth or survival advantage on cancer cells. Indeed, *ErbB2* gene amplification is one of the most frequent genetic events in breast cancer, resulting in the overexpression of HER2, which promotes cell proliferation predominantly through the activation of the mitogen-activated protein kinase (MAPK) pathway. However, *ErbB2* gene amplification can also induce breast cancer cell migration and invasion through the HER2-mediated activation of the Rho GTPases Rac1 and Cdc42, master regulators of cytoskeletal dynamics.^45,46^ Overexpression of fibroblast growth factor receptor 1 (FGFR1) due to amplification of the corresponding gene *FGFR1* promotes EMT and increases migration and invasion of H1581 NSCLC cells and DMS114 small-cell lung cancer cells in vitro by upregulating the expression of the transcription factor SOX2, one of the core operators of stemness and EMT.^47^ The amplification of wild-type *EGFR* and subsequent activation of the epidermal growth factor receptor (EGFR) contribute to the non-angiogenic invasive growth of glioblastoma in the patient-derived rat xenograft model probably through the induction of EMT and correlate with glioblastoma invasion in patients.^48^

Amplification of growth factor receptor genes is not the only way to induce cancer cell invasion and migration. Amplification of chromosome region 11q13, which encompasses genes encoding regulators of the actin cytoskeleton and cell motility (e.g., cortactin, cofilin and p21-activated kinase 1), occurs in 30–50% of head and neck squamous cell carcinomas (HNSCC).^49^ An in vitro study demonstrated that 11q13 amplification promotes the overexpression of cortactin, which binds to and activates the Arp2/3 actin-nucleating complex, leading to the increased migration and invasion of various HNSCC cell lines (UMSCC2, UMSCC19 and MSK921).^50^ By contrast, 11q13 amplification-driven overexpression of the *PPFIA1* gene, which encodes liprin-α1, a protein potentially involved in cell–matrix interactions, suppresses migration and invasion of FaDu HNSCC cells in vitro.^51^ These results indicate the presence of both positive and negative regulators of cell motility in this chromosomal region. Amplification of another chromosome region, 11q22.1–q22.2, is often found in oral squamous cell carcinomas and is associated with lymph-node metastasis. This amplification leads to overexpression of the *BIRC3* gene, the protein product of which—cellular inhibitor of apoptosis (cIAP)2—enhances the migration and invasion of SCC29B oral squamous carcinoma cells in vitro.^52^

Additional studies have shown that amplification of chromosome regions harbouring non-coding RNAs also triggers tumour cell migration and invasion. Gene-amplification-driven long non-coding RNA (lncRNA) SNHG17 promotes the migration of A549 and PC-9 NSCLC cells in vitro,^53^ whereas amplification of lncRNA PCAT6 is important for motility in HepG2 and SMMC-7721 hepatocellular carcinoma cells in vitro.^54^ Amplification and subsequent overexpression of miR-151 directly targets RhoGDIA, a putative metastasis suppressor, to promote the migration and invasion of Huh7 and SMMC-7721 hepatocellular carcinoma cells in vitro and the metastasis of SMMC-7721 cells.^55^ MiR-182, a member of the miRNA cluster in the chromosomal locus 7q31–34 that is frequently amplified in melanoma, stimulates the migration of SK-MEL-19 melanoma cells in vitro and increases the metastatic potential of B16F10 mouse melanoma cells.^56^

Box 1 A brief overview of the processes responsible for CIN CIN, one of the forms of genomic instability in tumours, is characterised by an increase in the rate of loss or gain of whole chromosomes or their fragments during cell division. CIN has a severe and complex impact on the genetic landscape of the tumour by affecting various oncogenes, tumour-suppressor genes and DNA-repair genes that drive cancer growth and progression. CIN promotes intratumoural heterogeneity and clonal evolution, giving cancer cells an advantage under selective pressure.^8^

Different mitotic events underlie CIN. Among them are cohesion defects, dysfunction in spindle assembly checkpoint, centrosome amplification and cytokinesis failure. Defects in DNA replication and repair, such as telomere dysfunction and replication stress, are also responsible for CIN. All these changes lead to chromosome missegregation during mitosis and pave the way to polyploidy, aneuploidy and diverse chromosomal rearrangements.^212,213^

The role of CIN in cancer growth and progression remains debatable. Some researchers consider CIN to be an early event in cancer, and some believe that CIN is simply a side effect of tumour growth.^8^ In any event, CIN is significantly associated with drug resistance and cancer progression.^8,10^

## Gene alterations

**Fig. 3:**
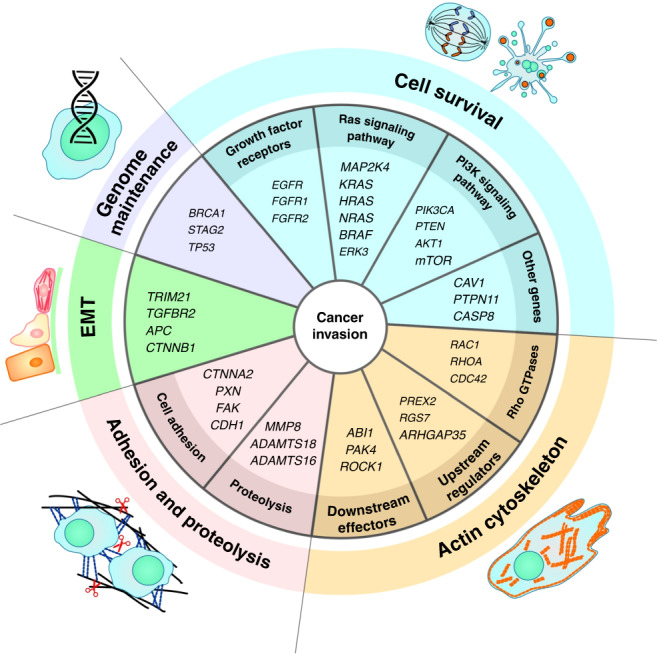
Gene alterations and cancer invasion. Various gene mutations can affect tumour cell migration and invasion. Genes responsible for genome maintenance are frequently mutated in cancers; however, only a few of them can influence tumour cell motility, the main player here being *TP53* and its diverse mutant forms. Alterations in genes that play a role in cell survival affect a variety of cellular processes and signalling pathways underlying cell migration. Mutations in genes encoding regulators of the actin cytoskeleton, adhesion, proteolysis and EMT directly influence the ability of tumour cells to migrate and invade.

In addition to harbouring chromosomal abnormalities, different cancers also contain an abundance of point mutations as well as gene insertions and deletions (indels). These gene alterations play a significant role in various stages of cancer metastasis, and invasion is no exception.^57^ Below, we outline those genes whose alteration affects the migration and invasion of tumour cells; they are divided into several groups, depending on their primary function (Fig. 3).

### Genes involved in genome maintenance

Genes involved in maintaining genome stability are often mutated in cancer. Not only do loss-of-function (LOF) mutations of these tumour suppressors contribute to the acquisition of a mutator phenotype by tumour cells, but they can also affect cancer cell migration and invasion. Mutations in *BRCA1* lead to dysregulation of the Ubc9/caveolin-l/vascular endothelial growth factor (VEGF)/SIRTl/oestrogen receptor (ER)-α axis, promote EMT and trigger the migration of HCC1937 triple-negative breast cancer cells in vitro.^58,59^ The *STAG2* gene, the protein product of which regulates centromere cohesion, is often mutated in various cancers. Most *STAG2* mutations are truncating and, as shown in the U2OS osteosarcoma cell line, the loss of this gene leads to increased EMT-associated tumour cell migration in vitro, coincident with decreased expression of E-cadherin and increased expression of N-cadherin.^60^

The best known ‘stabiliser’ of the genome and tumour suppressor, however, is p53. *TP53* is often mutated in a wide variety of tumours, from carcinomas and sarcomas to lymphomas and leukaemias.^61^ Loss of p53 due to LOF mutations often leads to increased activity of the transcription factors Snail and Twist1, decreased expression of E-cadherin and induction of EMT.^62–64^ In addition, p53 loss activates Rho GTPases to increase cell migration, as shown in mouse embryonic fibroblasts and A375P melanoma cells in vitro.^65,66^ However, loss of *TP53* might not always be sufficient to promote tumour cell invasion and metastasis, as shown in vivo in PVTT-1 hepatocellular carcinoma xenografts and transgenic mouse rhabdomyosarcoma model, indicating that gain-of-function (GOF) mutations of this gene are more potent activators of the metastatic cascade.^64,67^

GOF mutations in *TP53* cause an even more prominent effect on tumour cell invasiveness than do LOF mutations.^68,69^ Driver *TP53* GOF mutations often occur at codons 175, 248 and 273^61^ and endow the p53 protein with new abilities to regulate hundreds of different genes including other tumour suppressors.^70^ The mutants p53 R175H and R273H have been shown to bind to and inactivate the tumour suppressor p63 to form a mutant p53–p63 complex.^6^ This mutant complex suppresses Split and Hairy-related protein 1 (Sharp-1, a metastasis suppressor) and cyclin G2, and enhances TGF-β-mediated invasion and metastasis of MDA-MB-231 breast cancer cells in vitro and in vivo,^71^ as well as accelerates integrin recycling and activates signalling by the receptor tyrosine kinases EGFR and Met via Rab-coupling protein (RCP) in H1299 lung and MDA-MB-231 breast cancer cells.^72,73^ In these cancers, mutant p53 also promotes EGFR and Met signalling through the inactivation of a suppressor of invasion, Dicer ribonuclease,^74^ and enhances integrin and EGFR recycling and focal adhesion turnover by modulating components of the endosomal machinery.^75^ Inactivation of p63 by p53 mutants can also alter the expression of miRNAs involved in tumour cell migration. For example, mutant-p53-mediated upregulation of miR-155 leads to the increased migration and invasion of ZR-75-1 breast and H1299 lung cancer cells in vitro,^76^ and downregulation of tumour suppressor microRNA let-7i induced by the mutant p53–p63 complex leads to enhanced invasion of H1299 lung cancer cells in vitro.^77^ As demonstrated in H1299 lung cancer cells in vitro, formation of the mutant p53–p63 complex and the associated increase in cancer cell migration and invasion can be inhibited by the activating transcription factor 3 (ATF3) protein, which binds the mutant forms of p53 and thus facilitates p63 activation.^78,79^ It is important to note that the mutant p53–p63 complex and the mechanisms described above are not always required for the migration and invasion of tumour cells. Inactivation of Dicer ribonuclease mediated by mutant p53 can occur independently of the formation of the mutant p53–p63 complex.^74^

In addition, GOF mutant forms of p53 can trigger EMT via overexpression of Twist,^80^ stabilisation of Slug^81^ and also by acting on ZEB1.^82^ Mutant p53 can enhance the expression of the A1AT protein, which promotes EMT-associated migration and invasion of H2009 lung cancer cells in vitro, and drives invasion of H2009 cells in the chick chorioallantoic membrane in vivo assay.^83^ The p53 R248Q mutant activates the phosphorylation of Stat3, which results in the enhanced EMT-dependent migration of HCT116 colorectal cancer cells and H1299 NSCLC cells in vitro.^84^ Mice with p53 mutations in addition to the loss of another tumour suppressor, RB1, develop mammary tumours with EMT features.^85^

Numerous other studies have demonstrated the effect of GOF p53 mutations on a multitude of cell locomotion regulators.^69^ It should be noted, however, that p53 mutants can impact cell movement negatively as well as positively. For example, dominant-negative p53 mutants, such as R175H, R273H, R280K and R249S, can induce varying degrees of invasive potential in combination with the wild-type form of p53 in hTERT-HME1 (non-malignant) immortalised epithelial mammary cells. Thus, each of these p53 mutants may specifically affect the metastatic ability of cancer cells.^86^ In contrast, the p53 R248Q mutant negatively affects the migration of MDA-MB-231 breast and H1299 lung cancer cells in vitro and alters the distribution of MDA-MB-231 cells injected into zebrafish embryos, and contributes to mesenchymal–epithelial transition (the opposite of EMT).^87^ More research is therefore needed to understand the effects of different p53 GOF mutations on tumour cell motility and invasiveness.

### Genes involved in cell survival

Similar to genome-maintenance regulators, driver genes that modulate cell proliferation and survival are frequently mutated in different cancers. These genes encode growth factor receptors and components of Ras and phosphatidylinositol 3-kinase (PI3K) signalling pathways.

A significant effect on cancer cell migration and invasion is exerted by alterations in the genes encoding various growth factor receptors. In addition to the amplification of genes encoding various growth factor receptors (described above), point mutations and indels in these genes can also affect the motility of tumour cells. The *EGFR* L858R mutation enhances the migration and invasion of A549, H1299 and CL1–0 lung cancer cells in vitro.^88,89^ Notably, however, HOG glioma cells with this mutation migrate slower in vitro than cells with wild-type *EGFR*. Probably, this is due to the fact that *EGFR* oncogene does not initially provide a selective advantage for HOG cells, while the *EGFR* mutation negatively affects cell growth and migration.^90^ Another mutant, EGFRvIII, is characterised by the loss of two extracellular domains owing to the deletion of exons 2–7, which renders the mutant receptor constitutively active and unable to bind ligands. EGFRvIII promotes the migration and invasion of glioblastoma cells through the induction of proteases, integrin signalling and other mechanisms.^91–93^ The so-called ‘gatekeeper’ V561M mutation in *FGFR1* confers resistance to FGFR inhibitors, as well as promoting the mesenchymal phenotype and enhancing the ability of H1581 NSCLC cells to migrate and invade in vitro.^94^ Activating mutations in FGFR2 contribute to a loss of polarity and impair directional cell migration, but promote invasion of HEK-293FT endometrial cancer cells in vitro.^95^

Mutations in Ras-family GTPases are very common in various cancers and significantly affect tumour progression.^96^ *HRAS* Q61R and NRAS Q61R driver mutations induce EMT and enhance the migration of Nthy-ori 3–1 thyroid cancer cells and MCF10A breast epithelial cells, respectively.^97,98^ Driver mutations in *KRAS* at position G12 promote EMT via Wnt/β-catenin and TGF-β signalling pathways in the iKAP mouse model of colorectal cancer in vivo^99^ and in various pancreatic cancer cell lines in vitro and in vivo.^100,101^ Moreover, the *KRAS* G12 and *HRAS* G12 mutants can modulate the function of the Rho GTPases RhoA, Rac1 and Cdc42 through the Ras and PI3K signalling pathways in the Caco-2 colorectal cancer cell line in vitro and thereby mediate migration and invasion.^102^ Overexpression of *KRAS* G12V leads to a decrease of collective invasion of MCF10A cells.^103^

Mutations in genes encoding downstream effectors of Ras GTPases also affect the ability of tumour cells to move. The *BRAF* V600E driver mutation occurs in almost half of all melanoma cases and enhances the kinase activity of the BRAF protein.^104^ The V600E mutation induces the migration and invasion of WM3211 melanoma cells in vitro and the invasion of mouse melanoma in vivo by stimulating integrin signalling, actin protrusion formation and the expression of MMPs through activation of extracellular signal-regulated kinase (ERK)/MAPK.^105^ The *BRAF* V600E mutant also contributes to invasion of cancers other than melanoma. In thyroid cancer, the *BRAF* V600E mutant promotes cell movement through the nuclear factor (NF)-κB pathway as demonstrated in WRO and KTC-3 cell lines in vitro,^106^ or by mediating hypomethylation and subsequent overexpression of the gene encoding WAS/WASL-interacting protein family member 1 (WIPF1), as demonstrated in K1, OCUT1 and FTC133 cells in vitro and K1 cells in vivo.^107^ In the Caco-2 colorectal cancer cell line, *BRAF* V600E represses E-cadherin and enhances the activity of Rho GTPases.^102^ Other evidence also supports a role for *BRAF* mutants in EMT-associated tumour invasion.^108,109^

Mutations in the genes encoding ERK/MAPKs or MAPK/ERK kinases (MEKs) also modulate tumour cell movement. The *ERK3* L290P/V mutation promotes the migration and invasion but not proliferation of H1299 and A549 NSCLC cells in vitro.^110^ Loss of MKK4 protein due to *MAP2K4* LOF mutations enhances the invasion associated with peroxisome proliferator-activated receptor γ (PPARγ) of various lung cancer cell lines (344SQ, 393P and H2009) in vitro.^111^

*PIK3CA* and *PTEN*, which encode components of the PI3K signalling pathway, are among the most frequently mutated genes in various cancers.^112^ E545K and H1047R mutations in the p110 catalytic subunit of PI3K, which confer constitutive activity, have been shown to promote the migration and invasion of colorectal,^113^ gastric,^114^ cervical^115^ and breast cancer^116^ and HNSCC cells.^117^ In NOK and EPC1 HNSCC cell lines, the expression of mutant *PIK3CA* together with the downregulation of p120 catenin induces tumour invasion in vitro, including in 3D organotypic cultures, through an increase in the expression of MMPs.^118^ *PTEN* LOF mutations are observed in various cancers^119^ and contribute to EMT and the dissemination of tumour cells.^120,121^ For example, deletion of *PTEN* leads to increased collective invasion of MCF10A cells in contrast to *KRAS* G12V overexpression as mentioned above. Interestingly, the double PTEN and KRAS mutant cells show decreased collective behaviour, suggesting that KRAS dominates the collective migration phenotype.^103^ GOF mutations in *PTEN* are also known to modulate tumour cell movement. For example, the A126G mutant promotes the migration of PC3 prostate cancer cells in vitro.^122^

Mutations in the genes encoding AKT and mammalian target of rapamycin (mTOR), which are involved in the PI3K signalling pathway, are rare in cancers.^123^ However, mutant forms of these proteins can still contribute to cancer cell migration and invasion. The *AKT1* E17K mutation (0.6–2% frequency in NSCLC) enhances the migration and invasion of normal lung epithelial cells (BEAS-2B) by relocating the cyclin-dependent kinase inhibitor p27 into the cytoplasm from the nucleus and inhibiting RhoA signalling.^124^ The same mutated form of *AKT1* increases the migration and invasion of human mammary luminal (HMLER) but not myoepithelial (BPLER) cells.^125^ GOF mutations conferred by mutated *mTOR* occur with a frequency of no more than 1% for various types of cancer; some of these mutations (e.g., A1256G and G7076A) promote tumour cell migration and invasion in vitro.^126^

Mutations in other genes implicated in cell survival have also been reported to influence cell invasion. Retinoblastoma protein, encoded by *RB1*, is a well-known tumour suppressor that plays a role in controlling cell-cycle progression.^127^ Different mechanisms are involved in *RB1* loss, including LOF mutations and deletions.^127^ The knockdown-mediated loss of *RB1* expression in PC3, PC3-ML and LNCaP prostate cancer cells leads to the acquisition of an increased migratory and invasive capacity with decreased expression of E-cadherin in vitro.^128^ The loss of *RB1* in MYC-overexpressing mouse mammary epithelial cells promotes invasion in vitro and enhances the invasive phenotype in MYC-overexpressing xenograft tumours.^129^ Moreover, *RB1* suppression was demonstrated to stimulate collective invasion rather than single-cell invasion of basal-like breast carcinoma cells in vitro and in vivo. Importantly, Rb knockdown also induced expression of CD44, lymphovascular invasion, the release of circulating tumour cells and distant metastasis.^130^ The *CAV1* gene encodes caveolin-1, a component of caveolae—specialised plasma membrane invaginations that regulate cell proliferation and migration.^131^ Using the highly metastatic Met-1 mammary epithelial cell line, it was demonstrated that the *CAV1* P132L mutation, which occurs in 16% of breast cancers, promotes migration and invasion, and activates various signalling pathways involved in metastasis.^132^ The tyrosine phosphatase SHP2 (*PTPN11*) transmits signals from tyrosine kinase receptors and regulates cell proliferation. A GOF mutation in *PTPN11* that confers a D61G substitution enhances the migration and invasion of MDA-MB-231 and MCF-7 breast cancer cells in vitro and the metastasis of both cell lines in vivo through the activation of the Ras and PI3K signalling pathways.^133^ Caspases are best known as essential mediators of the apoptotic programme and cell survival, but mutations in the *CASP8* gene have been shown to accelerate migration and invasion of UM-SCC-47 HNSCC cells in vitro and their growth in vivo.^134^ Probably, it can be related to the catalytic and noncatalytic modes of action by which CASP8 influences cell adhesion and migration.^135^

### Actin cytoskeleton regulators

As mentioned above, Rho GTPases are key regulators of actin cytoskeleton remodelling. The best-studied Rho GTPases—Rac1 and RhoA—are often mutated in various types of cancer.^136^ *RAC1* is the third most frequently mutated gene in melanoma after *BRAF* and *NRAS*.^137^ The *RAC1* P29S driver mutation, which results from a C > T transition in response to UV damage, generates a more active form of Rac1. This mutant form is characterised by increased switching from the inactive, GDP-bound to the active, GTP-bound state, which enhances the interaction of Rac1 with its downstream effectors.^138^ The RAC1 P29S mutant promotes the migration of melanocytes^139^ and invasion of mouse embryonic fibroblasts in vitro.^140^ Although melanoma cells (104T cell line) with the *RAC1* P29S mutation form lamellipodia more actively, this mutant negatively affects the formation of invadopodia and invadopodia-dependent matrix degradation in vitro. This can indicate that *RAC1* P29S-harbouring melanoma cells have an enhanced migration, but attenuated invasion.^141^ *RHOA* is a driver gene in many cancers, such as T-cell lymphoma and gastric cancer.^142^ LOF mutants of *RHOA* (G17E, Y42C and Y42S) that are present in diffuse-type stomach cancers lead to the inactivation of RhoA–ROCK1 signalling and increased migration of MKN74 gastric tubular adenocarcinoma cells in vitro.^143^ Moreover, as shown in the orthotopic xenograft mouse model, MKN74 gastric cancer cells with *RHOA* mutations are more invasive and acquire immune resistance.^144^

Mutations of the genes encoding other Rho GTPases, such as Cdc42, Rac2, Rac3, RhoB and RhoC, are rare and their effect on tumour cell movement has not yet been characterised.^142^ However, as these Rho GTPases play an important role in the reorganisation of the actin cytoskeleton, their mutation probably also affects cancer cell migration.

The activity of Rho GTPases is positively regulated by Rho guanine nucleotide-exchange factors (GEFs) and negatively by Rho GTPase-activating proteins (GAPs);^145^ consequently, mutations in the genes encoding these Rho GTPase regulators significantly affect the migration and invasion of tumour cells. The *PREX2* gene, which encodes a RhoGEF, is often mutated in metastatic solid tumours.^146^ The *PREX2* S1113R mutant protein, present in patients with hepatocellular carcinoma, has been shown to promote the migration of Huh7 liver tumour cells in vitro.^147^ *RGS7*, which encodes a Rho GTPase-activating protein, is a tumour suppressor that is mutated in melanoma. The *RGS7* R44C mutation destabilises the protein, which thereby results in the enhanced motility of A375 melanoma cells in vitro.^148^ *ARHGAP35*, which encodes a negative regulator of Rho GTPases, is mutated in 15% of endometrial tumours. *ARHGAP35* GOF mutations (S866F and Δ865–870) contribute to random MDA-MB-231 breast cancer cell migration in vitro, which might promote the exploratory behaviour of tumour cells.^149^

Rho GTPases regulate downstream signalling effectors such as ROCKs, p21-activated kinases (PAKs), the SCAR/WAVE complex, LIM kinase (LIMK), cofilin and Arp2/3, which control actin cytoskeleton remodelling. Despite these effectors rarely being mutated in various cancers, it is logical to assume that mutations in their encoding genes, if they do occur, might affect the migration and invasion of tumour cells. Loss of the *ABI1* gene (which encodes a component of the SCAR/WAVE complex) leads to the induction of EMT and increased migration and invasion of RWPE-1 benign prostate epithelial cells in 2D and 3D in vitro systems.^150^ However, these results contradict the general consensus that overexpression of the SCAR/WAVE complex is associated with increased cancer invasion and poor prognosis, as outlined by Molinie and Gautreau.^151^ The E329K mutant of *PAK4* promotes the motility of PC3 prostate carcinoma cells in vitro,^152^ and GOF mutations in the *ROCK1* gene promote mouse embryonic fibroblast migration in vitro.^153^ However, it is important to note that, as mentioned above, mutations in downstream effectors of Rho GTPases are rare in cancer, and the dysregulation of these effectors in tumour cells is predominantly caused by other mechanisms.^154^

### Genes involved in cell adhesion and ECM proteolysis

Changes in cell adhesion and proteolysis of the ECM are inextricably linked to cell movement.^155^ Again, the genes underlying these processes are rarely mutated in cancers; however, experimental data indicate the importance of their potential mutations in the movement of tumour cells.

Integrins play a big role in cell adhesion, and changes in their expression promote cancer invasion.^155^ Although integrins are frequently dysregulated in various types of cancer, integrin mutations are poorly studied, especially in terms of their effect on tumour cell migration.^156^ The integrin β1 mutant T188I, which is found in poorly differentiated human squamous cell carcinoma of the tongue, enhances cell spreading (anchoring to the substrate) and actin cytoskeleton assembly, but does not promote migration or invasion of mouse keratinocytes in vitro.^157,158^ Note that cell spreading and cell motility are mechanistically different phenomena despite outward similarities.^159^ Integrin α7 is frequently inactivated in prostate tumours and leiomyosarcoma due to truncating mutations in the corresponding gene, and expression of wild-type *ITGA7* inhibits the migration of prostate cancer (PC3 and Du145) and SK-UT-1 leiomyosarcoma cells in vitro.^160^ Nevertheless, the effect of most integrin mutations on tumour cell migration and invasion remains unstudied.

Mutations in the genes encoding α-catenin (*CTNNA2* and *CTNNA3*) are characteristic of laryngeal squamous cell carcinoma and have been shown to promote tumour invasion of SCC-2 oral cancer cells in vitro.^161^ The adaptor protein paxillin (encoded by the *PXN* gene), a key component of focal adhesions, was mutated in up to 9.4% of NSCLC cases analysed by Jagadeeswaran et al.^162^ The most frequent mutation, A127T, enhances focal adhesion and lamellipodia formation in HEK-293 human embryonic kidney cells in vitro,^163^ and promotes the invasion of H522 NSCLC cells in vivo.^162^ EPHB6 is a receptor for ephrin-B ligands that modulates cell adhesion and migration. The *EPHB6* Q926R mutation activates RhoA through the induction, via JNK signalling, of cadherin-11 expression, and increases the invasion of A549 lung, Huh7 liver and A375P skin cancer cells in vitro.^164^ The deletion of exon 33 in the gene encoding focal adhesion kinase (*FAK*) confers a gain of function on the protein that enhances migration and invasion of MDA-MB-468 breast cancer cells in vitro.^165^ Onder et al. showed that truncating mutations in the *CDH1* gene, that lead to the expression of a dominant-negative protein, promote cell migration and growth of HMLER cells in vitro and in vivo, but to a lesser extent than the shRNA-mediated loss of E-cadherin.^166^ Other studies showed that *CDH1* mutations do not affect EMT or the motility of various breast cancer cell lines (MDA-MB-231, MCF-7, etc.) in vitro.^167,168^ All these data might indicate the cell-specific effect of CDH1 mutations.

Tumour cells must be able to degrade the ECM in order to penetrate the surrounding tissue and disseminate. It is therefore logical to assume that mutations in genes encoding proteases might alter the invasive potential of tumour cells. Similar to the situation regarding Rho GTPase effectors and integrins, most of the genes encoding various proteases, especially MMPs, are infrequently mutated in cancers; however, there are some data regarding the impact of their alterations on cancer cell migration and invasion. For example, mutations in the *MMP8* gene, often found in melanoma, enhance the migration of immortalised transformed human Mel-STR melanocytes in vitro and in vivo. Surprisingly, wild-type *MMP8* inhibits melanoma cell migration.^169^ Migration and invasion-suppressive role of MMP8 are also known in oral tongue squamous cell and breast carcinomas.^170,171^ Moreover, in breast cancer, MMP8 can prevent metastasis formation.^171^ The exact mechanisms of the suppressive effects of MMP8 are still unclear. Probably, MMP8 triggers migration- and invasion-suppressive molecular cascades through cleavage of various non-ECM substrates with specific regulatory functions.^172^ Similarly, mutations in the gene encoding a disintegrin-like and metalloproteinase domain with thrombospondin type 1 motifs (*ADAMTS18*) are potential drivers of melanoma and promote the migration of A375 melanoma cells in vitro and the metastasis of Mel-STR cells in vivo.^173^ Notably, however, evidence exists that mutations in protease genes can confer an inhibitory effect on the movement of tumour cells. Mutant forms of ADAMTS16 have been shown to inhibit the motility of A2780CP20 ovarian cancer cells in vitro and in vivo.^174^ Breast cancer-associated mutations in the *ADAM12* gene interfere with the intracellular trafficking of the corresponding protein and inhibit the migration of mouse embryonic fibroblasts in vitro.^175^ In general, proteases (especially MMPs) are considered as potential druggable targets in anticancer therapy,^176,177^ but whether their mutants can be therapeutically targeted is currently unknown, probably due to the fact that these genes are very rarely mutated in cancers. Furthermore, the enhanced migration of MMP8 mutant-immortalised melanocytes emphasises the need to assess the function of each MMP individually to define its precise role in cancer.

### EMT regulators

As demonstrated above, mutant forms of many oncogenes and tumour suppressors can modulate EMT through different mechanisms. But what about other regulators of EMT? Although mutations in genes encoding transcription factors that are involved in EMT (Twist, Snail, Slug and Zeb1) are known to be extremely rare in cancer,^178^ the activity of these transcription factors is regulated by other genes, mutations in which they can occur more frequently in various cancers. For example, mutations in the driver genes (*ADPGK* (encodes ADP-dependent glucokinase), *PCGF6* (polycomb group RING finger protein 6), *PKP2* (plakophilin 2), *NUP93* (nucleoporin 93) and *SLC22A5* (solute carrier family 22 member 5)) can affect EMT and promote MDA-MB-231 breast cancer cell migration in vitro.^179^ The gene encoding another EMT regulator, TRIM21, which promotes the proteasomal degradation of Snail and thereby suppresses migration and invasion, is rarely mutated in breast cancer (frequency <1%), but the R64Q mutation abrogates the ability of TRIM21 to mediate Snail degradation and thus promotes breast cancer cell invasion.^180^ GOF mutations in the TGF-β receptor II gene (*TGFBR2*) induce the re-localisation of E-cadherin from the cell membrane to the cytoplasm and overexpression of vimentin, and promote TGF-β signalling, migration and invasion of HSC-2 oral squamous cell carcinoma cells in vitro, contributing to aggressive cancer behaviour.^181^ Mutations in the genes that encode Smad transcription factor proteins, which are key mediators of TGF-β signalling, can promote TGF-β-mediated EMT.^182,183^ Furthermore, driver mutations in the *APC*, *CTNNB1* and *NOTCH1* genes, and other components of the WNT and Notch signalling pathways, contribute to EMT in various cancers.^184–186^

### Miscellaneous genes

As a consequence of mutation, genes that are not directly related to the regulation of cell movement can sometimes acquire new functions and thus promote cancer cell migration and invasion. Missense and nonsense mutations in the mitochondrial gene *ND6*, which normally encodes a subunit of NADH dehydrogenase (ubiquinone), promote migration and invasion of A549 lung adenocarcinoma cells in vitro, probably via the increased generation of reactive oxygen species.^187^ Activating mutations in the *GRM3* gene, which encodes a G-protein-coupled receptor, occur in melanoma and stimulate the migration of A375 melanoma cells in vitro, probably through phosphorylation of MEK.^188^

## Studying the effect of genetic alterations on tumour cell movement

Most current studies focus on the investigation of the effects of changes in various epigenetic determinants and gene expression on tumour cell migration and invasion, while the impact of genetic alterations on the ability of tumour cells to move undeservedly remains poorly studied. However, the irreversible nature of these genetic alterations might actually contribute more significantly to the invasion of tumour cells than other factors do.

### Current challenges

Many of the mutations described above occur in genes that regulate a wide array of cellular processes, and it is often difficult to separate their impact on migration and invasion from their influence on tumour formation—this can be a serious obstacle in studying the effect of genetic alterations on the motility of tumour cells. Moreover, it is hard to conclude whether tumour cell movement hinges upon certain mutations or other, non-genetic triggers. Another important issue is the need to identify mutational drivers of invasion and metastasis, both universal and specific for different types of cancer. Analysis of the studies discussed in this review shows that some genes (*TP53*, *EGFR* and *PIK3CA*) can be common for various cancers in terms of the effect of their mutations on tumour cell migration and invasion, whereas other genes are strongly specific for certain malignant tumours: for example, *RAC1* and *ADAMTS18* in melanoma, and *APC* in colorectal cancer (see Table 1). Even though some genes that are involved in cell motility are rarely mutated in cancers (such as downstream effectors of Rho GTPases and integrins), their mutations, no matter how infrequently they occur, might play a big role in driving cancer invasion. Moreover, each cancer is likely to be unique in its genetic landscape, and therefore mutational drivers important for invasion could vary significantly from tumour to tumour. Thus, further studies should be focused on the development of an atlas of mutational drivers of cancer invasion as an important step towards understanding the genetic subtleties that underlie tumour dissemination.

**Table 1:** Genetic alterations associated with migration and invasion of different cancer cells.

Cancer	Genetic alterations
Breast cancer	Chromosomal instability: polyploidy, *ESR1–YAP1* and *ESR1–PCDH11X* fusions, *ERBB2* amplification, LOH of 8p22 (*DLC1*) and LOH of 8p Gene alterations: *BRCA1*, *TP53*, *NRAS*, *PIK3CA*, *RB1*, *CAV1*, *PTPN11*, *ARHGAP35*, *FAK*, *CDH1*, *ADAM12*, *ADPGK*, *PCGF6*, *PKP2*, *NUP93*, *SLC22A5* and *TRIM21*
Colorectal cancer	Chromosomal instability: polyploidy, trisomy of chromosomes 5, 7, 13 and 18 Gene alterations: *TP53*, *KRAS*, *BRAF*, *PIK3CA*, *APC* and *SMAD4*
Prostate cancer	Chromosomal instability: *TMPRSS2–ERG* fusion and LOH of 8p22 (*DLC1*) Gene alterations: *PTEN*, *RB1*, *ABI1*, *PAK4* and *ITGA7*
Non-small-cell lung cancer	Chromosomal instability: *FGFR1* and *SNHG17* amplifications, and LOH of 8p22 (*DLC1*) and 1p32 (*TGFBR3*) Gene alterations: *TP53*, *EGFR*, *FGFR1*, *ERK3*, *MAP2K4*, *AKT1*, *PXN*, *EPHB6* and *ND6*
Melanoma	Chromosomal instability: miR-182 amplification Gene alterations: *TP53*, *BRAF*, *RAC1*, *RGS7*, *MMP8* and *GRM3*
Head and neck squamous cell carcinoma	Chromosomal instability: 11q13 amplification Gene alterations: *PIK3CA* and *CASP8*
Oral squamous cell carcinoma	Chromosomal instability: 11q22.1–q22.2 amplification Gene alterations: *TGFBR2* and *NOTCH1*

### Approaches to analysing mutational drivers of cancer invasion

Different approaches can be used to identify and study mutational drivers of cancer invasion. Metastatic mouse models of various cancers are an effective way to identify genetic alterations that contribute to tumour cell migration, invasion and metastasis.^189–192^ A 2017 study used a metastatic model of colorectal cancer to demonstrate that pronounced migration of tumour cells depends on the combined effect of mutations in *APC*, *KRAS*, *TP53* and *SMAD4*.^193^ It seems reasonable to analyse cancer genomes by focusing on the functionally significant mutations in genes that regulate critical processes in cell migration and invasion—for example, EMT, actin cytoskeleton remodelling, proteolysis and so on—and to validate their significance in vitro and in vivo. Another potential approach is to analyse the mutational landscape of tumour cells located in the invasive front, and to select for genetic alterations that are not present in the tumour core. For example, local invasion is a hallmark of malignant gliomas, making glioma cells a candidate model for finding drivers of cancer invasion.^194^ However, data also indicate that highly dynamic cells are present not only at tumour borders but also in the tumour core, as was demonstrated in NICD/p53^−/−^ mouse intestinal cancer^195^ and orthotopic human glioblastoma model,^196^ which significantly reduces the chance of finding mutations that drive cancer invasion when comparing the invasive front with the tumour core. In this case, it therefore seems reasonable to compare the mutational landscape of invasive and non-invasive tumour cells within the same tumour. Specific molecular markers could potentially be used to distinguish motile tumour cells from non-motile tumour cells in the primary tumour, and meticulous examination of the genomic landscape of such cells could uncover novel mutational drivers of cancer invasion. However, no effective and reliable markers to help identify truly motile tumour cells currently exist.^197^

**Fig. 4:**
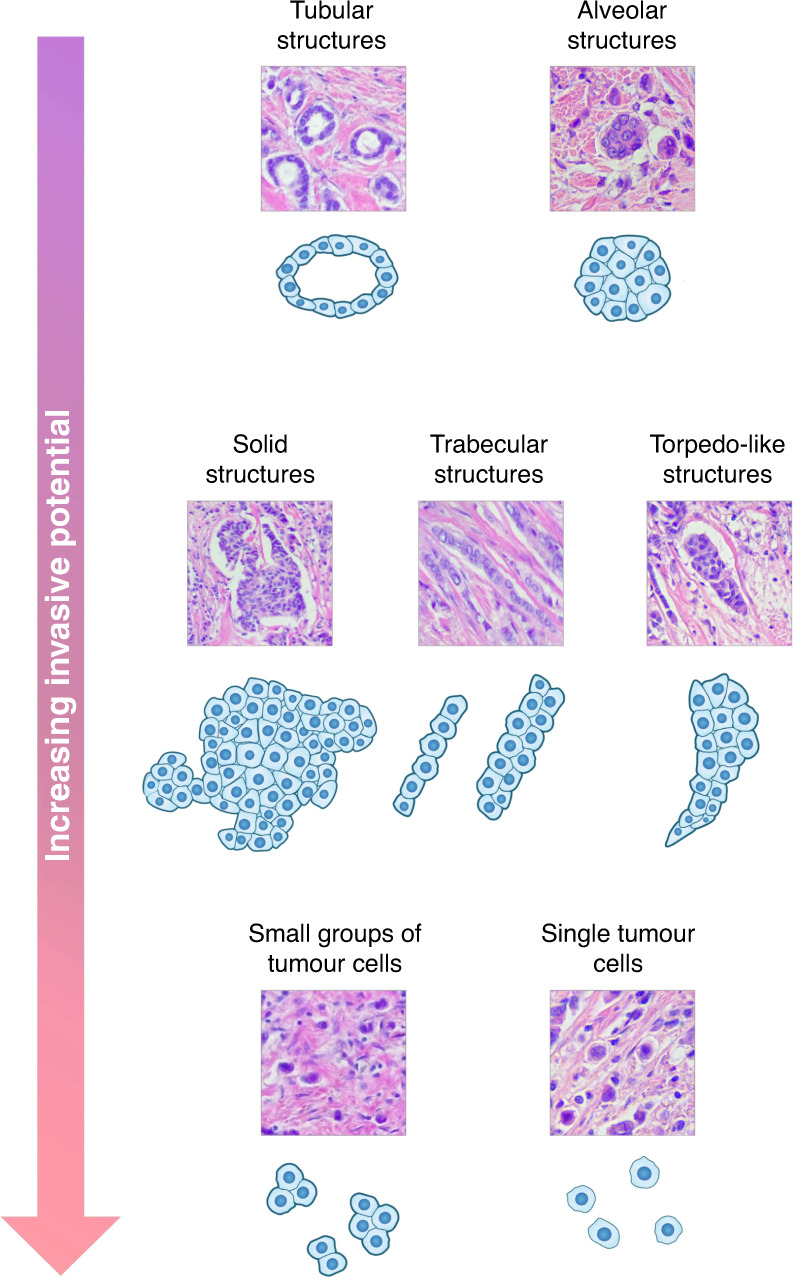
Intratumoural morphological heterogeneity of breast cancer as a model for studying the mechanisms of tumour cell invasion. Intratumoural morphological heterogeneity of invasive carcinoma of no special type, the common histological type of breast cancer, is represented by various types of architectural arrangements of tumour cells that significantly differ in the transcriptomic profile, namely in the expression of genes involved in EMT and enrichment of cancer invasion signalling pathways. Tubular and alveolar structures are similar in epithelial and mesenchymal gene expression patterns. Solid structures demonstrate an increase in mesenchymal markers but retain epithelial features. Trabecular structures display a pronounced mesenchymal phenotype and a dramatic decrease in epithelial traits. Small groups of tumour cells and single tumour cells show a strong mesenchymal phenotype and the significant enrichment of cancer invasion signalling pathways. Torpedo-like structures have been recently identified to be associated with breast cancer metastasis through the activity of kinesin-14 (KIF14), mitochondria-eating protein (Mieap) and ezrin (EZR) that are known regulators of tumour cell motility and invasion. However, the EMT degree of torpedo-like structures remains to be elucidated. Based on these data, it can be hypothesised that tubular and alveolar structures are less invasive, whereas solid, trabecular and torpedo-like structures, as well as small groups of tumour cells and single tumour cells, are highly invasive. In addition, considering the architectural features, solid, trabecular and torpedo-like structures, as well as small groups of tumour cells, can be attributed to collective cancer cell invasion, whereas single tumour cells—to individual cancer cell invasion.

In our studies, we have shown that the intratumoural morphological heterogeneity of invasive ductal carcinoma of the breast (now classified as invasive carcinoma of no special type) is a reflection of various patterns of tumour cell invasion. In particular, breast cancer cells can exist as single entities or be arranged in either small groups (2–5 cells) or multicellular structures: tubular, alveolar, solid, trabecular and torpedo-like structures (Fig. 4).^198,199^ Tubular and alveolar structures are transcriptionally similar and demonstrate a similar expression of epithelial and mesenchymal markers. Solid structures show an increase in mesenchymal traits but retain epithelial features. Trabecular structures, small groups of tumour cells and single tumour cells all display a pronounced mesenchymal phenotype and a dramatic decrease in epithelial traits, as well as significant enrichment of cancer invasion signalling pathways.^198^ The presence of alveolar and trabecular structures in breast tumours is associated with increased lymph-node metastasis^200,201^ and distant recurrence in patients treated with neoadjuvant chemotherapy.^202^ Distant metastases are also frequently detected in breast cancers with single tumour cells with epithelial-like morphology,^203^ and in breast cancers that express kinesin-14 (KIF14) and mitochondria-eating protein (Mieap), but lack ezrin (EZR) at the tips of torpedo-like structures.^199^ The nature of torpedo-like structures, e.g., their EMT features, remains to be elucidated; however, KIF14, Mieap and EZR proteins are known to be important regulators of tumour cell migration and invasion.^204–206^ Based on all these results, we assumed that tubular and alveolar structures show decreased invasive potential, whereas solid, trabecular and torpedo-like structures, as well as small groups of tumour cells and single tumour cells, are highly invasive. The intratumoural morphological heterogeneity of breast cancer is therefore an attractive model for detecting mutational drivers of tumour cell invasion—for example, by comparing the genomic landscapes of highly invasive and less invasive morphological structures. Moreover, comparative analysis of multicellular structures (e.g., solid, trabecular or torpedo-like structures) against single tumour cells might provide information regarding genetic mutations that are involved in collective and individual modes of cancer invasion.

## Conclusions and discussion

Different chromosomal and gene aberrations influence cancer cell migration and invasion. CIN affects cancer cell movement through mechanisms associated with polyploidy and aneuploidy, as well as with gene fusion and amplification. Gene alterations trigger or suppress the spread of cancer cells in several ways, by influencing genes that affect genome maintenance, cell survival, actin cytoskeleton remodelling, EMT, adhesion and proteolysis. Such genetic drivers are of particular interest as potential prognostic markers and targets for anti-metastatic therapy.

Indeed, some of the mutational drivers discussed in this review have already been established as potential targets for anticancer therapy—p53 hotspot mutations,^207^ *EGFR* mutations^208^ and PI3K p110α E545K and H1047R mutants.^209^ The main objective of anticancer therapy is to stop tumour growth and to kill cancer cells. However, another therapeutic approach, which is receiving ever-increasing interest, is to block the ability of tumour cells to invade and metastasise. Migrastatics are a novel class of anticancer drugs aimed at attenuating cancer cell migration by targeting the signalling pathways and downstream effectors that are involved in cell motility.^210^ The downside of these therapeutics is that they can be toxic for all types of moving cells—for example, fibroblasts, keratinocytes and leukocytes.^211^ In this regard, mutational drivers of cancer invasion could constitute especially interesting targets for migrastatics as these genetic alterations are present only in tumour cells. Nevertheless, this issue requires a great deal of further research.

Further studies are also needed to explore known genetic mutations as well as to identify novel ones that affect invasion in various cancers, and to understand the number, combination and sequence of potential driver mutations that are required to promote tumour cell movement. Moreover, it must be demonstrated whether such mutational drivers are capable of promoting the motility of tumour cells independently of other prometastatic factors, such as the tumour microenvironment, epigenetic alterations and gene expression changes, or if genetic alterations serve merely as a build-up for other determinants of cancer invasion and metastasis. One way or another, it is simply not enough to study the problem of cancer invasion and metastasis from one narrow point of view. An integrated approach, which combines the careful and considered examination of tumour cell motility at the genome, epigenome, transcriptome and proteome levels, is needed for a comprehensive understanding of cancer invasion and metastasis.

## Data Availability

Not applicable.
